# Retraction: Role of senescence and mitotic catastrophe in cancer therapy

**DOI:** 10.1186/1747-1028-7-15

**Published:** 2012-07-12

**Authors:** Richa Singh, Jasmine George, Yogeshwer Shukla

**Affiliations:** 1 Proteomics Laboratory, Indian Institute of Toxicology Research, Council of Scientific & Industrial Research, PO Box 80, MG Marg, Lucknow, 226001, India

## **Retraction**

This article [1] is retracted as it contains large amount of text that has been duplicated from other articles previously published. We apologize to all affected parties for the inconvenience caused.
